# Longitudinal study of calf morbidity and mortality and the associated risk factors on urban and peri-urban dairy farms in southern Ethiopia

**DOI:** 10.1186/s12917-023-03574-8

**Published:** 2023-01-20

**Authors:** Rahmeto Abebe, Temesgen Dema, Yohanis Libiyos, Woinshet Teherku, Alemayehu Regassa, Amene Fekadu, Desie Sheferaw

**Affiliations:** 1 Hawassa University, Faculty of Veterinary Medicine, P.O.Box 05, Hawassa, Sidama Ethiopia; 2 Ofa District Agricultural Development Office, Gasuba, SNNPRS Ethiopia; 3 Hobicha District Agricultural Development Office, Badda, SNNPRS Ethiopia; 4 Private Veterinarian, Addis Ababa, Ethiopia

**Keywords:** Calves, Ethiopia, Morbidity, Mortality, Prospective study, Risk factors

## Abstract

**Background:**

Calf morbidity and mortality are among the leading causes of economic losses on dairy farms around the world. Poor calf management practices exacerbate the problem in developing countries like Ethiopia. This prospective, longitudinal study was conducted on 70 selected dairy farms in southern Ethiopia with the aim of estimating calf morbidity and mortality rates, identifying the associated risk factors and determining whether the mortality rate is above economically tolerable levels. For this purpose, a total of 274 calves on 70 farms were followed up every two weeks from birth to six months of age for major clinical health problems and deaths.

**Results:**

The study found a morbidity rate of 13.2 cases and a mortality rate of 3.8 cases per 100 calf-months at risk in the study calves. The cumulative incidence of morbidity and mortality was also found to be 40.29% and 12.85%, respectively. Diarrhea was the leading cause of morbidity and mortality in calves, accounting for 71.3% and 62.1% of all morbidity and mortality, respectively. In a multivariable Cox regression analysis, the risk of morbidity was significantly (*p* = 0.022) higher in calves born to dystocia-affected dams (HR = 2.4) and on farms where dairy farming is the farmers’ secondary source of income (HR = 1.7). However, the risk of morbidity was significantly (*p* < 0.001) lower in calves older than three months (HR = 0.22), female calves (HR = 0.57), calves raised by farmers who had completed secondary school (HR = 0.26) or college education (HR = 0.30). Similarly, calves aged over three months (HR = 0.14), calves separated from their dams only after ingestion of colostrum (HR = 0.40) and calves owned by farmers who completed secondary school (HR = 0.08) or college education (HR = 0.13) all had lower mortality rates than other groups. On the other hand, calves born to cows with dystocia were 5.2 times more likely to die.

**Conclusion:**

The study concluded that calf morbidity and mortality rates in the study area are higher than economically tolerable levels and therefore it is recommended to raise awareness among farmers to improve calf management practices.

**Supplementary Information:**

The online version contains supplementary material available at 10.1186/s12917-023-03574-8.

## Introduction

Ethiopia has the largest livestock population in Africa, with an estimated 70.3 million head of cattle, of which 9.12% are calves under six months [1]. Dairy farming is one of the most significant segments of the livestock industry in Ethiopia, where calves are the future herd of a dairy farm. Dairy farming in urban and peri-urban regions is growing rapidly across the country due to increased urbanization and demand for milk and milk products, even though the extensive production system is still the country's leading method of livestock husbandry [2]. Urban and peri-urban dairies are semi-intensive to intensive production systems that maintain exotic and cross-bred cows with comparatively better management practices [3]. Although the dairy industry has grown significantly in recent years, it is reported to suffer from inefficient reproduction, low calf survival rate, high calf morbidity and mortality, and a high incidence of diseases such as mastitis, lameness, pneumonia, and ketosis [4].

Dairy farmers worldwide face recurring problems with calf morbidity and mortality [5], particularly in the tropics, where high temperatures and humidity promote the spread of infectious agents and make it difficult to effectively replace heifers [6]. Calf diseases result from the interplay of several factors, such as management practices in dairy farms, environmental conditions, infectious agents, and the calf itself [7]. Numerous diseases have been linked to calf morbidity and mortality. However, diarrhea in the neonatal period and pneumonia in older calves are known to be the major causes of calf morbidity and mortality [8]. Furthermore, several risk factors for calf morbidity and mortality have been identified, including inadequate or lack of colostral immunity, overcrowding and poor hygiene that increase the transmission of organisms, naive immune systems in newborns, stressors such as cold ambient temperature and frequent animal mixing, calf nutrition and calf vaccination status [9].

Over the years, several studies have been conducted on calf morbidity and mortality and associated risk factors in Ethiopia. According to a systematic review and meta-analysis of calf morbidity and mortality studies in Ethiopia, the prevalence of calf mortality ranges between 0.9% and 37%, with a pooled prevalence of 14.79% [10], while the prevalence of calf morbidity falls within the range of 22.8 to 66.7% [11]. The reported pooled mortality prevalence is above the economically tolerable levels of 3%–5% that can be achieved with good calf management and is defined as the minimum standard for the western production system [5]. Therefore, Ethiopia still has a long way to go to reduce calf mortality. The majority of the studies in Ethiopia employed a cross-sectional study methodology that provides insufficient detail on the extent of the problem and relevant risk factors. The other problem with previous studies in the country is the variation in the operational definition of morbidity rate, mortality rate and prevalence between studies. According to strict definitions, the word "rate" should only be used to describe metrics based on the idea of animal-time units. More specifically, it is a ratio where the denominator is the number of animal-time units that are at risk [12]. Furthermore, the majority of earlier studies only looked at certain known risk factors for calf morbidity and mortality.

The aim of this prospective, longitudinal study was therefore to estimate the morbidity and mortality rate in calves younger than six months using the standard method, to identify potential risk factors, and to determine whether the mortality rate in the study area is above the economically tolerable level.

## Materials and methods

### Study Area

This study was carried out on dairy farms in and around the towns of Hawassa, Arsi Negelle, and Wolayta Sodo. These areas were chosen for their relatively higher potential for dairy production in southern Ethiopia. In the study area, cattle are mainly kept for milk production to be sold to the city dwellers. Hawassa, the capital of the newly formed Sidama National Regional State, is located 273 kilometers south of Addis Ababa at 7° 3′ N latitude and 38° 28′ E longitude. It is at an altitude of 1708 meters above sea level. The city has an average annual rainfall of 800-1000 mm, and an average minimum and maximum temperature of 12.6 °C and 30.1 °C, respectively. Arsi Negele is located in the West Arsi zone of the Oromia regional state, 225 kilometers south of Addis Ababa. The town lies at 7°21'N latitude and 38°42'E longitude, approximately 2043 meters above sea level. The average annual minimum and maximum temperature is 10 and 25 °C respectively, while the annual rainfall ranges from 500 to 1000 mm. Wolayta Sodo is located at 6°54′N latitude and 37°45′E longitude, and is between 1600 and 2100 meters above sea level. The average annual rainfall in the town is between 450 mm and 1446 mm, while the average annual maximum and minimum temperatures are 26.6 and 11.4 °C, respectively.

### Study population and management practices

According to the Department of Livestock and Fisheries Development (2020) of each municipality, there are about 107 dairy farms in Hawassa, 35 in Arsi Negele, and 33 in Wolayta Sodo. Herd sizes ranged from 2 to 131 cattle, with an average of 7 cattle per herd. The cattle husbandry system in the study farms varied from semi-intensive to intensive husbandry system. In intensive farms, the cattle were kept in stalls the whole time and fed concentrates and roughages. The cattle on semi-intensive farms grazed outside during the day and were only given supplementary feed just before milking in the morning and evening. The study population consisted of calves under six months old on all dairy farms in the towns. In this study, calves were defined as cattle less than 6 months old [13]. In terms of breed, 10 (3.6%), 22 (8.1%) and 242 (88.3%) of the calves monitored were local zebu, Jersey and Holstein-Friesian zebu crosses, respectively. Artificial insemination (AI) was the most common method used by the dairy farmers to breed cows. However, if conception failed after the AI or the AI technician was late, all farms would use the bull service as a backup.

### Study design and sampling method

To achieve the aim of the study, a prospective longitudinal study was used. For this purpose, individual calves were identified and monitored regularly until the end of the study to detect the emergence of new cases of morbidity and mortality on the dairy farms. A questionnaire survey was also conducted during the study period to collect data at herd and calf level. Farm selection was based on herd size and farmer’s willingness to participate in the study. Consequently, 70 dairy farms with five or more cows were selected to increase the likelihood of finding at least one calf for follow-up. Based on these criteria, 23 farms from Hawassa, 26 from Arsi Negele and 21 from Wolayta Sodo were selected. These farms account for 40% of all farms in the areas. The total number of calves recruited for the study was 274.

### Data collection

#### Questionnaire survey

A semi-structured questionnaire was given to a dairy farm worker who was primarily responsible of animal managing during the first visit to the selected farm. The questionnaire was designed to collect data on a variety of topics that were divided into four categories: dairy farmer characteristics, management factors, calf factors, and dam factors. Dairy farmer data included age, sex, education level, and whether dairy farming is a primary or secondary source of income. The management parameters considered were the housing arrangement of the calving facility, the feeding arrangement, the cleanliness of the calf pen, umbilical health care, the time and method of colostrum feeding, the weaning age, the mixing of calves of different ages or calves with cows in the barns, and other factors. The calf-related data were sex, breed and age. The parity number and the delivery status (normal/dystocia) were taken into account as dam factors. Dystocia was defined in this study as parturition that lasted longer than six hours and required assistance, whereas normal parturition was defined as parturition that occurred with little or no assistance.

#### Monitoring of calves

All newborn calves were identified at each farm visit by their ear tags, if present, or other tags used by the investigator, and were monitored up to 6 months of age. Two calves, 30 and 45 days old, purchased from other farms were also included in the study. Each calf in the study was observed every two weeks until the end of the study. All calf illnesses and calf deaths occurring between visits were documented in a standard format designed separately for each farm. A preliminary diagnosis of clinical health problems observed during a regular visit was made by the investigators through a clinical examination while the farm attendants were asked to list and describe any health problems that emerged between visits. In addition to regular visits, emergency visits have also been made in response to calls from dairy farmers. Calves were excluded from follow-up at 6 months of age. If a calf was lost to follow-up, the date and reason for the loss was recorded. For the purposes of this study, morbidity was defined as any illness with observable clinical signs or symptoms manifested by calves that ultimately results in death or warrants therapeutic intervention during follow-up. Any observed calf death after 24 hours of life, regardless of cause, was referred to as mortality. Coughing, nasal discharge, and breathing difficulties were used to diagnose pneumonia, while loose manure that persisted for two days or more was used to define diarrhea.

### Statistical analysis

Data obtained through questionnaire survey and prospective observation of calves were stored, filtered and coded in Microsoft Excel 2007 spreadsheets and transferred to Stata version 14.2 (Stata Corp. TX USA, 2006) for statistical analysis. The outcome variables in this study were morbidity and mortality rates, which were recorded as “1” if the relevant event occurred or “0” otherwise. Morbidity and mortality events were estimated as crude incidence rates. The incidence rate is defined as the rate at which an event occurs per unit of animal-time at risk [12]. Therefore, the morbidity or mortality rate was calculated as the number of morbidity events or deaths that occurred during the observation period divided by the total risk periods. The periods at risk, reported here as calf- months at risk, represent the total number of months that the calves were present during the study period without experiencing disease-related events or remaining alive. A rate was reported as per 100 calves-months at risk. The cumulative incidence of morbidity or mortality was also estimated using the Kaplan-Meier (K-M) life table for comparison with other studies. The cumulative incidence was defined as the probability that a disease event will occur in a calf (for morbidity) or the probability that a calf will die (for mortality) during a follow-up period. About 19 variables mentioned in Table 4 were considered as explanatory variables in the statistical analysis.

The log-rank test was used to test the null hypothesis that there are differences between groups of categorical predictors in the probability of an event (illness or death) and to determine whether or not the categorical predictor should be included in the final model or not. A p-value cut off of 0.25 from the log-rank test was used as a standard to select a variable for the multivariable model. The risk factors associated with calf morbidity and mortality rate were assessed using a multivariable Cox proportional hazards regression model. Collinearity between the predictors was checked using tabulation and correlations, using gamma values between -0.6 to 0.6 as a benchmark in each analysis [12]. The final model was built using stepwise backward elimination of non-significant variables. Potential confounders were controlled at every step of the model building process. A variable was deemed a confounder if coefficients of the remaining variables changed by 20%. The Schoenfeld and scaled Schoenfeld residuals were used to evaluate the assumption that the hazards in the Cox proportional hazard model are proportionate or constant across time with different predictors or covariate levels [14]. The hazard rate (HR) with its 95% confidence interval was used to report the measures of effect of different predictors on the outcome variable.

## Results

### Description of the dairy farms

Men owned 75.7% of all farms, compared to women who owned only 24.3%. According to the level of education, 34.3%, 24.3% and 34.3% of the dairy farms were run by farmers who completed their elementary, secondary and college education, while 7.1% were run by farmers who never had received formal training. Dairy farming was the primary source of revenue for 62.86% of farmers, whilst for 37.1% it was only a secondary source. The majority (84.3%) of dairy farms used an intensive management system, where the cattle were always housed and fed concentrates and roughages. The remaining 15.7% of dairy farms used a semi-intensive management system, where the cattle were allowed to graze outside during the day but only received supplemental feeding in the morning and evening just before milking. The majority (91.4%) of dairy farms have no maternity facilities. Colostrum was given to newborn calves on 77.1% of farms immediately after delivery whereas on 22.9% of farms six hours later. On 41.4% of farms, calves were fed manually, as opposed to 58.6% that allowed them to consume colostrum directly from their mothers. It is feasible for calves to mix on 68.6% of the farms because they were not kept according to their ages and sizes, which is thought to make it simpler for infectious agents to spread. Calves and dams were housed in the same barns on 64.3% of the farms. Only 7.1% of the farms provided newborn calves with routine care for the umbilical cord. The majority of farmers (80%) routinely cleaned their calf quarters, in contrast to 20% of farms where cleaning was inconsistent. Routine cleaning consisted of daily removal of manure and dirty straw/bedding (on the farms using bedding) and washing the concrete floor with water.

### Calf morbidity and mortality rate

Of the 274 calves that were observed in this study, 101 (36.9%) showed signs of one or more clinically apparent health problems, while 29 (10.6%) died due to various causes. Disease incidences and deaths in the study calves were recorded up to 4 months of age, after which no morbidity or mortality events were noted. As a result, the data were truncated after 4 months and accordingly the overall morbidity and mortality rates are calculated as 13.2 cases per 100 calf months and 3.8 cases per 100 calf months at risk, respectively (Table 1). A total of 122 calves were lost to follow-up during the observation period before the end of the study. Among them, 111 (90.98%) were male and 11 (9.02%) were female. The sales of calves, particularly male calves, for slaughter and feedlot was the main cause of calf withdrawal, whereas the female calves were sold as replacement stock to other farms. The majority (86.1%) of calf withdrawal was noticed after 3 months of age (after weaning) (Table 2).

**Table 1:** Morbidity and mortality rate of calves on dairy farms in southern Ethiopia based on geographical location

Geographical location	No. calves at risk	No. cases	Time at risk (months)	IR /100 calf month	95% CI for IR
**Morbidity**					
Arsi Negele	46	16	129.50	12.4	7.6, 20.2
Hawassa	114	37	352.95	10.5	7.6, 14.5
Wolaita Sodo	114	48	284.00	16.9	12.7, 22.4
Total	274	101	766.45	13.2	10.8, 16.0
**Mortality**					
Arsi Negele	46	10	129.5	7.7	4.2, 14.4
Hawassa	114	10	352.95	2.8	1.5, 5.3
Wolaita Sodo	114	9	284	3.2	1.6, 6.1
Total	274	29	766.45	3.8	2.6, 5.4

*IR* Incidence rate

**Table 2:** Cumulative incidence of all-cause morbidity and mortality in calves under 6 months of age in southern Ethiopia

Age interval in months	Calves at risk	No. cases	No. censored	Cumulative incidence	95% CI
**Morbidity**					
[0-1)	274	23	8	8.52	5.74, 12.54
[1-2)	243	46	7	26.09	21.22, 31.83
[2-3)	190	14	6	31.62	26.36, 37.64
[3-4)	170	18	56	40.29	34.42, 46.76
[4-5)	96	0	74	40.29	34.42, 46.76
[5-6)	22	0	22	40.29	34.42, 46.76
**Mortality**					
[0-1)	274	8	8	3.05	1.54, 6.00
[1-2)	258	15	38	9.54	6.44, 14.02
[2-3)	205	4	16	11.52	8.04, 16.40
[3-4)	185	2	72	12.85	9.06, 18.07
[4-5)	96	0	74	12.85	9.06, 18.07
[5-6)	22	0	22	12.85	9.06, 18.07

Note: The age interval in each row includes the lower value but not the upper value

### Cumulative incidence of morbidity and mortality

In addition to calculating the morbidity and mortality rate, we also attempted to determine the cumulative incidence of all-cause morbidity and mortality in the study calves using the K-M life table approach. The cumulative incidence of all-cause morbidity and mortality was thus determined to be 40.29% and 12.85% respectively, during the follow-up period. In other words, the probability of a calf remaining disease-free or surviving at the end of the follow-up period was 59.71% and 87.15%, respectively. The cumulative incidence of morbidity and mortality increased steadily up to 4 months of age, but no disease development or deaths were recorded thereafter (Table 2). To better visualize the cumulative survival probability of calves from birth to six months of life, the same data were also presented using the K-M curve (supplementary file 1).

### Causes of morbidity and mortality

Diarrhea was the leading cause of morbidity and mortality accounting for 71.3% and 62.1% of all morbidity and mortality, respectively. Pneumonia was the second most frequent cause of morbidity, whereas unexplained causes were the second most frequent cause of death. Table 3 lists the other prevalent causes of morbidity and mortality.

**Table 3:** Major causes of morbidity and mortality in the 274 calves monitored in southern Ethiopia

Causes	Morbidity (N = 101)		Mortality (N = 29)	
	No. of cases	Percentage (%)	No. of cases	Percentage (%)
Diarrhea	72	71.3	18	62.1
Pneumonia	19	18.8	4	13.8
Septicemia	3	2.97	-	-
Joint illness	5	4.95	-	-
Navel illness	6	5.94	-	-
FMD	1	0.99	-	-
Mechanical injury	2	1.98	1	3.4
Ring worm	1	0.99	-	-
Unknown causes	-	-	6	20.7

### Risk factors for calf morbidity and mortality

#### Univariable analysis

In this study, the effect of 19 distinct host and management factors on calf morbidity and mortality was assessed using the Log-rank test, and potential predictors for the final multivariable analysis were selected based on the results. From these, the variables with *p*-values less than 0.25 were selected for the multivariable analysis of the risk factors, totaling 10 variables for morbidity and 9 variables for mortality (Table 4).

**Table 4:** Univariable analysis of risk factors for calf morbidity and mortality using Log-rank test

**No**	**Variable**	**Morbidity**		**Mortality**	
		x^2^	*p*	x^2^	*p*
	Location (Arsi Negele/Hawassa/Wolaita Sodo)	2.57	0.2771	6.02	0.0493
2	Cal sex (Male/Female)	9.55	0.002	3.0	0.0834
3	Calf breed (Jersey/HF cross)	1.51	0.4697	0.13	0.9349
4	Calf age (≤3/>3 yr)	35.92	0.0000	10.18	0.0014
5	Calving difficulty (Yes/No)	9.28	0.0023	23.04	0.000
6	Dam’s parity (Primiparous/Multiparous)	0.09	0.7702	3.01	0.0825
7	Management system (Intensive/Semi-intensive)	0.00	0.9829	0.10	0.7507
8	Farmer’s education (No formal edu/primary/secondary or college edu)	10.53	0.0145	14.03	0.0025
9	Herd size (≤10/>10)	0.02	0.8996	0.02	0.8907
10	Calving facility (Yes/No)	5.84	0.0156	0.09	0.7621
11	Calf housing (Individual/Group)	1.29	0.2561	0.61	0.4333
12	First time calves ingested colostrum (≤6 hrs/>6 hrs)	2.04	0.1530	0.02	0.8775
13	Time calves are separated from dams (Immediately/after sucking colostrum)	0.08	0.7726	7.76	0.0053
14	Method of colostrum administration (Sucking/Hand-fed)	1.12	0.2892	1.86	0.1727
15	Umbilical care (Yes/No)	5.04	0.048	0.06	0.8125
16	Mixing of calves of different ages (Yes/No)	2.94	0.086	0.14	0.7082
17	Mixing of calves and cows in the barn (Yes/No)	0.04	0.8441	1.25	0.2639
18	Hygiene (Good/Poor)	4.2	0.1225	3.18	0.2038
19	Dairying as a primary income source (Yes/No)	8.26	0.004	1.85	0.1735

#### Multivariable analysis

**Calf morbidity rate**: The incidence rate of calf morbidity was found to be significantly (p < 0.05) impacted by calf age, calf sex, calving difficulty, whether dairy farming is a primary or secondary source of income, and the farmer's educational status in the final multivariable Cox regression analysis model. Accordingly, compared to their counterparts, calves over 3 months old (HR = 0.22), female calves (HR=0.57), and calves on farms owned by farmers with secondary education (HR = 0.26) or a college degree (HR = 0.30) had a lower risk of morbidity. In contrast, calves born to dystocia-affected cows had a higher risk of morbidity (HR = 2.4), as did calves from dairy farms owned by farmers whose main source of income is not dairy farming (HR = 1.7) (Table 5). The final model was tested for the proportional hazards assumption and found not to violate the assumption (global test: Chisq = 6.08; df = 5; *p* = 0.2985).

**Table 5:** Risk factors for calf morbidity rate based on multivariable Cox regression analysis Table 5. Risk factors for calf morbidity rate based on multivariable Cox regression analysis

Variables	Category	HR	P >z	95% CI for HR
Calf age	< 3 mon	Ref		
	>3 mon	0.22	<0.001	0.12 - 0.39
Calf sex	Male	Ref		
	Female	0.57	0.018	0.36 - 0.91
Calving difficulty	No	Ref		
	Yes	2.4	0.022	1.13 - 5.03
Dairy farming as a primary source of income	Yes	Ref		
	No	1.7	0.029	1.05 - 2.65
Farmer’s education	No formal Edu	Ref		
	Primary	0.61	0.184	0.30 - 1.26
	Secondary	0.26	0.007	0.10 - 0.69
	College	0.30	<0.001	0.16 - 0.58

*Ref* Reference, *HR* Hazard Ratio

**Calf mortality rate**: The final multivariable Cox hazard regression analysis model revealed that among the assessed potential risk factors, calf age, calving difficulty, time of calf separation from dams, and educational level of dairy farmers all had a statistically significant (p<0.05) impact on the calf mortality rate on dairy farms in southern Ethiopia. In comparison to their counterparts, the mortality risk was lower in calves older than 3 months (HR = 0.14), calves taken from their dams only after they had sucked colostrum (HR = 0.40), and calves from farms owned by farmers who had completed secondary education (HR = 0.08), or tertiary education (HR = 0.13). Compared to calves born normally, calves from cows with dystocia exhibited a higher mortality risk (HR = 9.3) (Table 6). The final model was tested for the proportional hazards assumption and found not to violate the assumption (global test: Chisq = 4.82; df = 4; *p* = 0.1853).

**Table 6:** Risk factors for calf mortality rate based on multivariable Cox regression analysis

Variables	Category	HR	P >z	95% CI for HR
Age of calves	< 3 mon	Ref		
	>3 mon	0.14	0.002	0.04 - 0.48
Calving difficulty	No	Ref		
	Yes	9.3	<0.001	3.08 - 28.02
Time of separation of calves from dams	Immediately after birth	Ref		
	After sucking colostrum	0.40	0.028	0.18 - 0.90
Farmer’s education	No formal Edu	Ref		
	Primary	0.28	0.061	0.07 - 1.06
	Secondary	0.08	0.019	0.01 - 0.65
	College	0.13	0.001	0.04 - 0.41

*Ref* Reference, *HR* Hazard Ratio

## Discussion

The present study has shown that the crude morbidity and mortality rate of calves in the study areas is 13.2 cases per 100 calf months and 3.8 cases per 100 calf months at risk, respectively. The present study also revealed that the cumulative incidence of all-cause morbidity and mortality was 40.29% and 12.85%, respectively. With the exception of one study [15], there are methodological variations in the estimation of the morbidity or mortality rate in earlier studies in Ethiopia. The current morbidity and mortality rate is comparable to the crude morbidity and mortality rate reported by Hordofa et al. [15], which was 13.81 cases/100 calf months and 4.12 cases / 100 calf months, respectively. The cumulative incidence of morbidity in the current study is substantially lower than the range of 50.12 to 66.7% reported from different parts of the country [15–18]. It exceeds the cumulative incidence of 29.3% to 34.1% found in other studies [11, 19], nevertheless. Similarly, the cumulative mortality incidence found in the current study is higher than that reported by Megersa et al. [19] and Tora et al. [11], which corresponds to 9.3% and 8.64%, respectively, but lower than the incidence ranges reported in other investigations (20.04-30.7%) [15–18, 20]. Compared to studies outside Ethiopia, the current cumulative mortality incidence is higher than estimates from Europe, which vary between 1 and 9% [8, 21, 22], but within the range (13.0-30.0%) reported from other African countries [23]. Variations in morbidity and mortality rates between studies can be attributed to a range of calf- and herd-level risk factors, agroecology, case definition, age of the calves, study design, sample size, and study methodology [24].

The current study assessed a wide range of potential risk factors for calf morbidity and mortality. Accordingly, multivariable Cox regression analysis revealed that calf age, calf sex, calving difficulties, whether dairy farming is a primary or secondary source of income, and dairy farmers' educational level were risk factors for calf morbidity, while calf age, calving difficulties, the time calves were separated from their dams, and educational status were risk factors for calf mortality.

In comparison to calves under 3 months old, calves older than 3 months had a 78% (HR = 0.22) reduced risk of morbidity and an 86% (HR = 0.14) lower risk of mortality. In keeping with our findings, studies from Ethiopia and elsewhere have also demonstrated that calf morbidity and mortality rates are much higher in the early life of calves, particularly during the first month of a calf’s life [5, 8, 16, 17, 21]. In general, the relatively higher risk of morbidity and mortality in young calves found in previous and current studies implies that dairy farmers need to pay due attention and provide calves with the best possible health care at an early age.

The current study found that female calves had a 43% (HR = 0.57) reduced risk of morbidity than male calves. Although the difference was not statistically significant, female calves also had a lower mortality rate than male calves. This is probably related to the better management and health care services provided for female calves in the farms because female calves are considered future replacement stocks on farms and are of greater economic significance. Male calves often get less attention when it comes to feeding, medical care and other things. Although data from all farms are lacking, some dairy farmers we interviewed responded that raising male calves is not profitable and therefore they usually sell them at a young age for slaughter or fattening. As a result, they ignore concerns about feeding and other management issues, potentially leading to higher morbidity and mortality rates. Therefore, there is a need to raise dairy farmers’ awareness in order to change their attitude and reduce morbidity and mortality rates in male calves. Similar findings have also been reported by other studies conducted in Ethiopia and abroad [19, 25, 26].

Calves born to dams with dystocia had a 2.4 and 9.3 times higher risk of morbidity and mortality, respectively, compared to normally born calves. This result is in line with what has been described in previous studies [15, 21, 27, 28]. Due to the high possibility of contamination during delivery and delayed suckling or decreased colostrum intake, assisted delivery increases the risk of disease and mortality [28]. Furthermore, due to stress during delivery, adrenocorticotropic hormone is released. This hormone stimulates the adrenal cortex to produce and secrete more cortisol, leading to immunosuppression and increasing calves’ vulnerability to numerous pathogens [29]. Dystocia can also affect the vitality of new-born calves. According to Campler et al. [30] calves with low vitality are less inclined to stand up and suckle, which prevents the passive transfer of maternal antibodies.

In the current research area, dairy farming was the primary business for 62.86% of the farmers but it was only a sideline for 37.1% of the farmers. The risk of morbidity was 1.7 times higher on dairy farms where dairy farming is the secondary source of income for the farmer. This is related to the fact that farmers devote less time caring for the calves since they spend much of their time engaged in other activities that would give them a better income.

While some dairy farms at the current research sites leave newborns with their mothers for a few hours to suckle as much colostrum as possible, some dairy farms take calves from their mothers shortly after birth and offer colostrum six hours later. The risk of mortality was 60% (HR = 0.40) lower in calves that were allowed to stay with their mothers and consumed colostrum immediately than in calves that were immediately removed from their mothers and bottle-fed colostrum with some delay. The most likely explanation for this is that the calves, which were left with their mothers after birth, were able to ingest sufficient colostrum to protect them adequately from infectious agents. In contrast, calves that are taken away from their mothers and bottle-fed may not get enough colostrum at the right time. Failure to receive timely passive transfer of maternal antibodies may be the main reason for a higher risk of morbidity and mortality in these calves. It is recommended that calves be fed 10% to 12% of their body weight with high quality colostrum (3–4 L for a Holstein calf) within 1 to 2 hours after birth to successfully transfer passive immunity [31]. Furthermore, studies have shown that providing calves with 2 to 3 L of colostrum 5 to 6 hours after the first feeding optimizes the transfer of passive immunity, reduces morbidity and improves average daily weight gain in dairy calves [32].

In the current study, the educational level of the farmers had a significant (p<0.05) influence on the morbidity and mortality rates of the calves. The risk of morbidity was shown to be 74% (HR = 0.26) and 70% (HR = 0.30) lower in farms owned by farmers who completed secondary and college education, respectively as to compared to farms owned by farmers without a formal education. The risk of mortality was also decreased on the farms of farmers who completed their secondary school and college education by 92% (HR = 0.08) and 87% (HR = 0.13), respectively. This is most likely related to more effective calf management practices used on farms run by farmers with a higher level of education. This is evidenced by the fact that in comparison to calves from farmers without formal education or with only primary school, all farms of farmers with college degree and 91% of farms of farmers with secondary education provided the new-born calves with colostrum immediately after birth, separated them from their dams only after ingestion of colostrum and housed them in cleaned stalls.

The most prevalent disease syndrome and the main factor in calf deaths in the current study was diarrhea, followed by pneumonia. It accounted for 71.3% of all morbidity and 62.1% of all mortalities. Similar to the current data, other authors in Ethiopia have identified that diarrhea and pneumonia are the two most significant health issues affecting calves [11, 15–18, 33]. In studies conducted outside Ethiopia, the two disease conditions were likewise included as the primary and secondary causes of calf morbidity and mortality [34, 35]. The incidence of diarrhea was more pronounced in very young calves, possibly due to the sensitivity of newborn calves to diarrhea-causing agents and inadequate transfer of passive immunity as a result of not providing enough colostrum in a timely manner [31, 32]. Poor hygiene management of feeding equipment, the calving environment, and the calf pen can also contribute to the occurrence of diarrhea as a cause of calf morbidity and mortality [7]. Diarrhea is one of the major cause of economic losses for cattle farmers worldwide due to high morbidity and mortality in calves, especially in the first few weeks of life. In order to manage diarrhea, and its impact on the calf’s growth and performance in the future, it is crucial to determine the root causes. Therefore, future calf morbidity and mortality studies in the country should take into account identifying the underlying causes of diarrhea. The present study also found that 20.7% of calf deaths were due to unknown causes. Because the study was based solely on observation and physical examination, we could not identify the specific causes. Therefore, future studies must apply specific and highly sensitive diagnostic methods in order to identify the primary infectious and noninfectious causes of calf deaths on current dairy farms and elsewhere.

## Conclusions

The present study has revealed high morbidity and mortality rates of calves on dairy farms in southern Ethiopia. The reported mortality rate is higher than what can be achieved through sound management and is therefore not economically justifiable. This indicates short-term and long-term negative impacts on dairy production and replacement stocks. The study also found that diarrhea, pneumonia, and other undiagnosed diseases are the most common causes of morbidity and mortality in calves in the study area. Male calves, calves under three months of age, calves from dams with dystocia, calves from farms where dairy farming is the secondary source of income for farmers, and calves from farms run by farmers without a formal education all had a significantly increased risk of morbidity. Similarly, calves under 3 months of age, calves born to dams with dystocia, calves separated from their mothers shortly after birth, and calves reared on farms run by farmers without formal training had a significantly increased risk of death. Therefore, it is crucial to educate dairy farmers about good calf management practices, particularly the timely provision of sufficient colostrum and the proper management of dystocia cases as well as calves born to cows with dystocia, in order to reduce calf morbidity and mortality rates and, ultimately, improve the profitability of dairy production in the study area.

## Supplementary Information

**Additional file 1:Fig 1.** Kaplan-Meir Survival Estimate of Calf morbidity from Birth to 6 Months. **Fig 2.** Kaplan-Meir Survival Estimate of Calf mortality from Birth to 6 Months.

## Data Availability

All data generated or analyzed during this study are included in this article and are available from the corresponding author upon reasonable request.
