# Supplementary material for: Longitudinal study of calf morbidity and mortality and the associated risk factors on urban and peri-urban dairy farms in southern Ethiopia
Source: BMC Vet Res. 2023 Jan 20;19:15. doi: 10.1186/s12917-023-03574-8 (PMC9854030; doi:10.1186/s12917-023-03574-8)
Supplement: Supplementary file 1 — Additional file 1:Fig 1. Kaplan-Meir Survival Estimate of Calf morbidity from Birth to 6 Months. Fig 2. Kaplan-Meir Survival Estimate of Calf mortality from Birth to 6 Months. [file 12917_2023_3574_MOESM1_ESM.docx]

Fig 1. Kaplan-Meir Survival Estimate of Calf morbidity from Birth to 6 Months

Fig 2. Kaplan-Meir Survival Estimate of Calf mortality from Birth to 6 Months
